# Correlations among traditional Chinese medicine constitutions, sleep quality, and cancer-related fatigue in patients with breast cancer

**DOI:** 10.3389/fpubh.2026.1823328

**Published:** 2026-05-28

**Authors:** Yi Lu, Yanmin Xue, Xiangmin Xu, Zhongning Zhou, Xiyu Wang, Yingtao Meng, Mingxia Li

**Affiliations:** 1 Nursing School, Shandong University of Traditional Chinese Medicine, Jinan, Shandong, China; 2 Cancer Hospital of Shandong First Medical University Shandong Cancer Institute, Jinan, China

**Keywords:** breast neoplasms, fatigue, mediation analysis, medicine, Chinese traditional, sleep quality

## Abstract

**Purpose:**

To investigate the status of Traditional Chinese Medicine (TCM) constitution, sleep quality, and Cancer-Related Fatigue (CRF) in breast cancer (BC) patients and explore the relationships among these three factors, thereby providing a basis for reducing the level of CRF in individuals with BC.

**Methods:**

BC patients were enrolled from two Tertiary Level-A Hospitals in Shandong Province, China, using convenience sampling. Data were collected using the General Information Questionnaire, the Self-Measurement Scale for the Classification and Determination of TCM constitutions, the Pittsburgh Sleep Quality Index (PSQI), and the Piper Fatigue Scale (PFS).

**Results:**

A total of 320 breast cancer patients were included, predominantly exhibiting unbalanced constitutions (72.19%). Correlation analysis indicated that a balanced constitution was negatively associated with cancer-related fatigue (CRF) (*r* = −0.461, *P* < 0.01), In contrast, Qi deficiency, Yang deficiency, phlegm-dampness, dampness-heat, Qi stagnation constitutions, and poorer sleep quality were positively correlated with CRF (all *P* < 0.01). Multiple linear regression identified Qi deficiency, Yang deficiency, and Qi stagnation as significant independent predictors of CRF (*P* < 0.05). Furthermore, structural equation modeling revealed that sleep quality partially mediated the relationship between CRF and these three constitutions, with mediation proportions of 22.47%, 22.52%, and 37.5%, respectively.

**Conclusions:**

The level of CRF in patients with BC is moderate and multifactorial. Therefore, medical personnel can alleviate CRF by addressing patients' unbalanced constitutions to improve sleep quality.

## 1 Introduction

Breast cancer (BC) is one of the most prevalent malignant diseases among women. According to data from the National Cancer Institute (NCI), it ranks first in both incidence and mortality rates among all female malignancies, making it one of the most common cancers globally, a finding confirmed by multiple countries (1, 2). The Standard Treatment Guidelines for BC report indicates that in 2020, China recorded approximately 416,000 new BC cases and over 117,000 deaths from the disease, with both incidence and mortality rates showing an upward trend (3, 4). Currently, BC treatment primarily relies on chemotherapy and radiotherapy. While advanced therapeutic techniques have improved patient survival rates, prolonged treatment cycles often lead to systemic and local side effects (5). Cancer-related fatigue (CRF), as one such complication, profoundly impacts multiple dimensions of patients' quality of life and serves as an independent risk factor for reduced survival (6). Research indicates that CRF affects nearly 65% of cancer patients. Over two-thirds report symptoms lasting at least 6 months, while one-third experience persistent fatigue years after treatment (7). This fatigue is particularly pronounced among BC patients, with over 25% reporting it for 10 years or longer post-diagnosis (8). A substantial proportion of breast cancer patients, ranging from 34% to 90%, experience significant and enduring CRF (9). This condition often constitutes a severe and disabling symptom that profoundly impairs quality of life and functional capacity.

CRF is not merely a physiological issue but is also closely intertwined with sleep quality, exhibiting a bidirectional relationship. Research indicates that reduced sleep quality may increase the risk of BC (10), and sleep quality serves as a significant predictor of CRF in cancer patients. Improving sleep quality, deepening sleep, and promoting relaxation can effectively alleviate the severity of individual CRF (11).

Concurrently, Traditional Chinese Medicine (TCM) constitution theory, as a rapidly evolving research field, has demonstrated significant relevance in social epidemiological investigations. In TCM theory, constitution refers to a relatively stable, inherent characteristic formed in an individual throughout their life through a combination of genetic factors and environmental influences. This characteristic determines an individual's ability to adapt to environmental stimuli and their susceptibility to certain diseases. Traditional Chinese Medicine classifies constitutions into nine types: one balanced type, known as the “harmonious constitution,” and eight unbalanced types, including qi deficiency, yang deficiency, yin deficiency, phlegm-dampness, damp-heat, blood stasis, qi stagnation, and special constitution (12). A large-scale retrospective medical record study revealed strong associations between TCM constitution types and most diseases, including BC and insomnia (13), Recent research further indicates a significant correlation between TCM constitution and sleep quality, providing a basis for understanding and intervening in sleep from a constitutional perspective (14). A TCM constitution reflects an individual's holistic state, serving both to prevent constitution-related diseases and to buffer symptoms and signs of illness. However, existing research has primarily focused on examining the relationship between TCM constitution types and single health issues, such as sleep, depression, or specific diseases (15–17), or treating cancer-related fatigue as an isolated symptom (18). Currently, there is a lack of empirical research systematically examining the interplay among TCM constitution types, sleep quality, and cancer-related fatigue in breast cancer patients within a unified framework. This research gap limits our ability to develop integrated, individualized intervention strategies grounded in the holistic principles of TCM.

Given this, the present study investigates the correlations among TCM constitution, sleep quality, and CRF among BC patients, aiming to provide theoretical foundations and practical guidance for clinical research and intervention implementation.

## 2 Methods

### 2.1 Design

This study is a cross-sectional investigation aimed at exploring the correlations among TCM constitution, sleep quality, and CRF in BC patients and validating the mediating role of sleep quality in the relationship between TCM constitution and CRF.

### 2.2 Participants and setting

The study employed convenience sampling to recruit hospitalized BC patients from the breast surgery and medical oncology departments of two Grade A tertiary hospitals in Jinan City, Shandong Province, between February and September 2022. All participants provided informed consent and signed the consent forms. A total of 341 questionnaires were distributed. After excluding incomplete or invalid responses, 320 valid questionnaires were recovered, yielding a valid response rate of 93.84%.

The inclusion criteria were as follows: (1) Female patients aged ≥18 years; (2) Pathologically confirmed diagnosis of primary BC; (3) Completion of primary treatment such as surgery, chemotherapy, or radiotherapy; (4) Being conscious with basic literacy and communication skills, providing informed consent, and voluntarily participating. The exclusion criteria were as follows: (1) Critically ill or in terminal disease stage; (2) Concurrent malignant tumors; (3) History of psychiatric disorders or cognitive impairment; (4) Severe dysfunction or complications involving major organs such as heart, brain, or lungs.

This study was approved by the Ethics Committee of the Affiliated Hospital of Shandong University of Traditional Chinese Medicine (Approval No. 2022-0017). All procedures performed in this study involving human participants were in accordance with the ethical standards of the institutional and/or national research committee and with the 1964 Helsinki declaration and its later amendments.

### 2.3 Instruments

**General information and clinical characteristics survey questionnaire:** Designed by researchers based on relevant domestic and international literature, the questionnaire comprises sections on sociodemographic data and clinical characteristics, including age, marital status, number of children, educational attainment, disease duration, surgical approach, and pathological type.

**Classification and determination of physique in Chinese medicine (ZYYXH/T157-2009):** Developed by the China Association of Chinese Medicine (19), this scale comprises nine subscales: Harmonious Constitution, Qi Deficiency Constitution, Yang Deficiency Constitution, Yin Deficiency Constitution, Phlegm-Dampness Constitution, Damp-Heat Constitution, Blood Stasis Constitution, Qi Stagnation Constitution, and Special Constitution. Each subscale contains 6 to 8 items, totaling 60 items (20). Each item is scored on a 1–5 Likert scale, with Cronbach's α ranging from 0.70 to 0.80 (21). As an industry standard, this scale has been widely used in large-scale population surveys and clinical studies in China. This study assigned patients' TCM constitution types based on the highest score in the dominant constitution subscale.

**Pittsburgh Sleep Quality Index (PSQI):** Developed by American scholars Buysse et al. (22), this scale assesses participants' sleep status over the preceding month. The scale comprises seven dimensions: subjective sleep quality, sleep latency, sleep duration, habitual sleep efficiency, sleep disturbances, use of sleep aids, and daytime dysfunction. It consists of 19 items, each scored 0–3, yielding a total score range of 0–21. The Cronbach's α is 0.83. Higher scores indicate poorer sleep quality. This study employed the Chinese version of the PSQI developed by Liu et al. (23), with a Cronbach's α of 0.842.

**Piper Fatigue Scale (PFS):** Developed by American scholars Piper et al. (24), this scale aims to measure patients' perceptions of CRF. The scale comprises 23 items across four dimensions: physical fatigue, behavioral fatigue, emotional fatigue, and cognitive fatigue. Each item is rated on a scale of 1 to 10, with higher scores indicating more severe CRF. The Cronbach's α is 0.97. This study utilized the Chinese version of the PFS developed by So et al. (25), with a Cronbach's α of 0.91.

### 2.4 Data collection data analysis

Data from this study were analyzed using IBM SPSS Statistics for Windows, version 26.0 (IBM Corp., Armonk, NY, USA). The normality of continuous variables was assessed using the Shapiro-Wilk test, complemented by an evaluation of skewness and kurtosis values. All variables approximated a normal distribution, and parametric tests were therefore applied. Frequency and percentage distributions were used to describe patients' sociodemographic and clinical characteristics. Independent-samples *t*-tests or one-way ANOVA were used to compare CRF scores among patients with varying demographic and clinical features. A Pearson correlation analysis was conducted to examine the relationships among TCM constitution, sleep quality, and the total CRF score.

To identify independent factors influencing CRF, a multiple linear regression analysis was conducted. Variables statistically significant in univariate analysis, along with TCM constitution types and total sleep quality scores, served as independent variables, while total CRF scores acted as the dependent variable. Entry criteria were set at *P* < 0.05, and exclusion criteria at *P* > 0.10. To examine the mediating role of sleep quality between specific TCM constitution types and CRF, the Bootstrap method (5,000 repeated samples) was used to test the mediation effect. A mediating effect was considered significant if the 95% confidence interval for the indirect effect did not include zero. All statistical analyses used *P* < 0.05 as the criterion for statistical significance.

## 3 Results

### 3.1 General information and clinical characteristics of study participants

The 320 BC patients were all female, mostly aged 50–59 years (40%); most participants were married (95%), and most of them had one child (50.6%), their education level was mainly junior high school (33.8%), and most of them were engaged in other jobs (35.9%), and 213 (66.6%) were from cities and towns, their family income (yuan/month) mostly was most frequently above 5,000 yuan (39.4%), and their medical payment method was mainly employee health insurance (45.9%).

Clinical characteristics: Most of the 320 BC patients (56.6%) were diagnosed within the past year. The primary operation type was non-breast-conserving surgery (70.9%). In terms of pathological type, most of the patients had predominantly infiltrative BC (94.4%), and stage II was the predominant disease stage (41.2%).

### 3.2 Current status of TCM constitution, sleep quality, and CRF in BC patients

It was found that the majority of BC patients had unbalanced constitutions, with 231 patients (72.19% of the total sample) and 27.81% of all individuals having balanced constitutions; notably, some individuals exhibited a mixed constitution, with two or more constitution types. Further statistical analysis was based on the TCM constitution with the highest conversion score; the total sleep quality score of the BC patients was 9.46 ± 4.47, and among the seven dimensions, the daytime dysfunction dimension had the highest score (1.77 ± 0.95), followed by the sleep onset latency dimension (1.70 ± 0.99); BC patients mainly experienced moderate CRF (60.00%), and the total CRF score was 94.63 ± 31.78; among the four dimensions, the sensory fatigue dimension had the highest score (4.76 ± 1.80). The behavioral fatigue dimension had the lowest score (3.83 ± 1.78).

### 3.3 Univariate analysis of CRF in BC patients

Different social-demographic data for BC patients were used as independent variables, and the total CRF score was used as the dependent variable. Independent-samples *t*-tests and one-way ANOVAs were used. The results revealed that the number of children, education level, family income level, disease stage, and surgical method significantly affected CRF in BC patients (*P* < 0.05), as shown in Table 1.

**Table 1:** Effects of demographic and clinical characteristics on CRF in BC patients (*n* = 320, Mean ± SD).

Characteristics	Groups	Score	t/F	*P*
Age	18–39 years	88.17 ± 43.32	0.871	0.457
	40–49 years	96.78 ± 31.76		
	50–59 years	93.38 ± 30.08		
	≥60 years	97.60 ± 27.29		
Marital status	Unmarried	63.00 ± 33.96	1.50	0.216
	Married	94.89 ± 31.84		
	Divorced	104.25 ± 32.22		
	Bereaved	82.40 ± 15.31		
Number of children	0	62.00 ± 25.42	4.201	0.006
	1	93.41 ± 30.60		
	2	95.43 ± 31.90		
	3 or more	108.74 ± 34.37		
Education level	Primary school and below	104.82 ± 33.69	3.081	0.028
	Junior high school	94.93 ± 29.07		
	High school/Secondary school	93.00 ± 33.13		
	University and above	87.88 ± 31.06		
Careers	Peasant	94.20 ± 30.82	0.032	0.992
	Self-employed person	95.86 ± 31.61		
	Enterprise and public institution	95.11 ± 34.78		
	Else	94.36 ± 31.35		
Family residence type	Countryside	95.50 ± 34.40	2.684	0.728
	City and town	94.19 ± 30.45		
Family income level (monthly)	Less than 1,000 Yuan	112.48 ± 33.96	3.532	0.015
	1,000–3,000 Yuan	91.10 ± 29.54		
	3,000–5,000 Yuan	93.42 ± 31.60		
	More than 5,000 Yuan	93.42 ± 31.68		
Medical expense payment method	Self-supporting	84.37 ± 28.07	0.544	0.653
	Residents' medical insurance	97.54 ± 34.55		
	Employee medical insurance	94.44 ± 30.99		
	New Agricultural Cooperative Society (NACS)	94.40 ± 31.86		
Time of diagnosis	≦1 year	91.61 ± 31.70	1.281	0.281
	1–5 years	99.02 ± 31.56		
	5–10 years	97.74 ± 32.83		
	≥10 years	96.82 ± 30.73		
Surgical procedure	Breast-conserving surgery	85.76 ± 31.24	0.034	0.001
	Non-breast-conserving surgery	98.27 ± 31.35		
Pathological type	Infiltrative	94.81 ± 31.94	0.157	0.684
	Non-infiltrative	91.67 ± 29.63		
Disease stage	Phase I	71.33 ± 24.32	21.181	0.000
	Phase II	94.05 ± 26.91		
	Phase III	100.34 ± 32.96		
	Phase IV	121.75 ± 32.45		

### 3.4 Analysis of the correlations among TCM constitution, sleep quality, and CRF in BC patients

Pearson correlation analysis was performed to examine the relationships among TCM constitution, sleep quality, and CRF scores in BC patients; the results are detailed in Table 2.

**Table 2:** Correlation analysis of TCM constitution, sleep quality, and CRF in BC patients (*n* = 320, *r*).

Characteristic		Total score for CRF	TCM constitution	Total sleep quality score
Total score for CRF		1	-	-
TCM constitution	Balanced constitution	−0.461^***^	1	-
	Qi deficiency constitution	0.393^***^		
	Yang deficiency constitution	0.237^***^		
	Yin deficiency constitution	0.233^***^		
	Phlegm-dampness constitution	0.263^***^		
	Dampness-heat constitution	0.169^**^		
	Blood stasis constitution	0.160^**^		
	Qi stagnation constitution	0.297^***^		
	Inherited a special constitution	0.023		
Total sleep quality score	0.365^**^	Balanced constitution	−0.426^***^	1
		Qi deficiency constitution	0.312^***^	
		Yang deficiency constitution	0.159^**^	
		Yin deficiency constitution	0.249^***^	
		Phlegm-dampness constitution	0.217^***^	
		Dampness-heat constitution	0.107	
		Blood stasis constitution	0.246^***^	
		Qi stagnation constitution	0.339^***^	
		Inherited a special constitution	0.049	

^***^p < 0.001, ^**^p < 0.01.

### 3.5 Multiple linear regression analysis of CRF in BC patients

Multiple linear regression analyses were conducted with the total CRF score as the dependent variable, and the statistically significant sociodemographic variables and clinical characteristics, balanced constitution, Qi deficiency constitution, Yang deficiency constitution, phlegm-dampness constitution, Qi stagnation constitution, and sleep quality score as the independent variables. The assignment of the independent variables and the setting of the dummy variables are shown in Table 3. The results of the multiple linear regression analyses revealed that the number of children, disease stage, balanced constitution as the benchmark, Qi deficiency constitution, Yang deficiency constitution, Qi stagnation constitution, and TCM sleep quality were the factors influencing CRF in BC patients, as shown in Table 4.

**Table 3:** Assignment of independent variables for factors influencing CRF in BC patients (*n* = 320).

Variable	Assignment method
Number of children	None = 0, 1 = 2, 2 = 2, 3 or more = 3
Household income level	Less than 1,000 yuan = 1; 1,000 yuan- 3,000 yuan = 2; 3,000 yuan−5,000 yuan = 3; 5,000 yuan and above = 4
Educational attainment	Elementary school and below = 1, middle school = 2, high school/junior college = 3, college and above = 4
Disease stage	Phase I = 1 Phase II = 2 Phase III = 3 Phase IV = 4
Surgical procedure	Breast-conserving surgery = 1, non-breast-conserving surgery = 2
Chinese medicine	Based on the balanced constitution: Qi deficiency constitution 01000, Yang deficiency constitution 00100, phlegm-dampness constitution 00010, Qi stagnation constitution 00001.
Sleep quality	Measured value

**Table 4:** Multiple linear regression analysis of CRF in BC patients (*n* = 320).

Variable	*B*	Standard error	Standardized regression coefficient	*t*	*P*
Constant	27.422	11.792		2.325	0.021
Number of children	7.125	2.676	0.147	2.663	0.008
Disease stage	13.517	2.015	0.352	6.709	0.000
Qi deficiency constitution	20.293	6.454	0.176	3.144	0.002
Yang deficiency constitution	13.057	4.562	0.170	2.862	0.005
Qi depression constitution	9.816	4.573	0.134	2.146	0.033
Sleep quality	2.242	0.414	0.309	5.422	0.000

R = 0.581, R^2^ = 0.337, adjusted R^2^ = 0.311, F = 12.875, p < 0.001.

### 3.6 Mediating effects of sleep quality in BC patients on TCM constitution and CRF

Bootstrap tests were used to test the mediating effect of sleep quality. The results revealed that the bootstrap 95% CIs of the direct effects of Qi deficiency constitution, Yang deficiency constitution, and Qi depression constitution on the effect of CRF and the mediating effect of sleep quality did not contain 0, indicating that sleep quality partially mediated the effects of Qi deficiency constitution, Yang deficiency constitution, Qi depression constitution, and cancer-caused fatigue, with mediating effects of 5.495, 3.518, and 7.190, accounting for 22.47%, 22.52%, and 37.48% of the total effect, respectively, as shown in Table 5.

**Table 5:** Analysis of the mediating effect of sleep quality on the relationship between TCM constitution and CRF.

Effect type	TCM constitution	Efficiency value	SE	95% confidence interval		
				Lower limit	Upper limit	Relative affect value
Aggregate effect	Qi deficiency constitution	24.455	7.2000	0.001	10.272	-
	Yang deficiency constitution	15.620	5.0919	0.002	5.589	-
	Qi stagnation constitution	19.182	4.8980	0.001	9.534	-
	Qi deficiency constitution	18.960	7.0768	0.008	5.020	77.53%
Direct effect	Yang deficiency constitution	12.102	4.9897	0.016	2.273	77.48%
	Qi stagnation constitution	11.992	5.0263	0.018	2.091	62.52%
	Qi deficiency constitution	5.495	2.3712	1.555	10.848	22.47%
Indirect effect	Yang deficiency constitution	3.518	1.6420	0.736	7.111	22.52%
	Qi stagnation constitution	7.190	2.2195	3.282	11.918	37.48%

## 4 Discussion

### 4.1 CRF in BC patients is at a moderate level

This survey revealed that the total CRF score was 94.63 ± 31.78 in BC patients, and most (60%) experienced moderate CRF, which was similar to the results of the study by Wang et al. (26). Among the four dimensions of CRF, the sensory fatigue dimension had the highest average score (4.76 ± 1.80), and the behavioral fatigue dimension had the lowest (3.83 ± 1.78). The reasons may be related to disease symptom clusters, and related studies have shown that CRF and sleep disorders, pain, and depression form symptom clusters that show correlations and synergistic effects (27, 28). Owing to pain, BC patients are often unable to sleep at night or even have their days and nights switched, resulting in depression, and sleep disorders further lead to poor recovery of physical strength, a decline in physical function, and physical and mental fatigue, ultimately exacerbating the feeling of fatigue. Second, in this study, most BC patients were under 60 years of age, and the diagnosis was made within a relatively short period. The physical fitness of young and middle-aged patients was better than that of older patients. In addition, patients with a shorter diagnosis time may not have received many forms of treatment, meaning that their work and study, social interactions, hobbies, or other aspects of life have not yet been affected, and the degree of behavioral fatigue is low.

In the future, health care professionals should recognize the symptoms of BC patients as early as possible and intervene in time to reduce the burden of CRF caused by symptom clusters; another study shows (29) that exercise therapy can improve the level of CRF in cancer patients, and health care professionals should formulate targeted exercise programs, such as aerobic exercise (30) or its combination with anti-group training (31) for patients during different periods of treatment.

### 4.2 Multiple factors influence CRF in BC patients

#### 4.2.1 The degree of CRF varies with different TCM constitutions

The results of this study revealed that the BC patients mainly had unbalanced constitutions (72.19%). The three unbalanced constitutions with higher transformation scores were Qi depression constitution, Yang deficiency constitution, and blood stasis constitution, similar to the results of the study by Zhang et al. (32). Among these constitutions, the balanced constitution was negatively correlated with the total CRF score, and the higher its transformation score, the lower its total CRF scores. The balanced constitution is positive and healthy. Patients with this constitution are easy-going and cheerful, have strong adaptive ability, and can carry out anticancer treatment with an open and receptive state of mind; thus, it is negatively correlated with several dimensions of CRF and is an important protective factor against CRF in BC patients.

Qi deficiency constitution, Yang deficiency constitution, phlegm-dampness constitution, dampness-heat constitution, and Qi stagnation constitution are positively correlated with the total CRF score, which shows that the higher the transformation scores of the above five constitutions are, the higher the total CRF score will be. BC patients with Qi deficiency constitution tend to be lethargic and talk less; BC patients with Yang deficiency constitution tend to be introverted and depressed; and BC patients with phlegm-dampness constitution, dampness-heat constitution, and Qi stagnation constitution tend to experience qi stagnation and blood stasis and depression, and their sensitivity to disease is increased, which leads to more negative emotional experiences.

#### 4.2.2 Worse sleep quality is associated with higher levels of CRF

CRF and sleep disorders are significant clinical symptoms in cancer patients. This study revealed that the total score and seven dimensions of sleep quality were significantly and positively correlated with the total score and four dimensions of CRF. The poorer the sleep quality and the higher the score, the greater the level of CRF in BC patients, which is consistent with the results of most studies (33, 34). A study has found that (35) sleep problems in BC patients usually coexist with various somatic and psychological problems. Insomnia problems are considered important predictors of CRF in cancer patients and play a role in this process. The reasons may be that sleep is an important way to restore physical strength and self-repair, and that good sleep can promote physical recovery through anabolic processes such as protein and tissue synthesis (36). In contrast, poor sleep quality disrupts patients' biological rhythms and reduces immune function. Long-term sleep disorders put patients into a vicious circle of deteriorating health and mental conditions, which, in turn, worsens CRF (37).

In recent years, mind-body therapies (MBRTs) (38, 39) have been developed and shown to improve the sleep quality of cancer patients. Therefore, health care professionals should actively develop mind-body therapies and select appropriate treatments for BC patients, in line with relevant guidelines or norms, to improve their sleep quality. Furthermore, simple and accessible non-pharmacological interventions rooted in TCM principles, such as hot water footbath therapy, have demonstrated effectiveness in improving sleep quality among cancer patients (40). This aligns with the TCM constitutional perspective of this study, suggesting that such therapies may indirectly alleviate CRF by regulating the body's yin-yang balance and improving sleep. Future research could explore integrating constitution-based TCM nursing interventions—including footbath therapy—into comprehensive CRF management for BC patients.

### 4.3 Mediating role of sleep quality in the relationship between TCM constitution and CRF in BC patients

Although the relationship between sleep disorders and CRF has been well documented in the literature (34), our study does not merely replicate this finding; rather, it aims to elucidate a potential mechanism of action—namely, that TCM constitution may be an upstream, fundamental influencing factor. Our mediation model offers a new perspective on understanding “why” certain patients are more prone to suffering from both sleep disorders and CRF. The results of this study confirmed that sleep quality partially mediated the relationship between Qi deficiency constitution and CRF, and between Yang deficiency constitution and CRF. Between Qi stagnation constitution and CRF (β = 5.495, 3.518, and 7.109), with the mediating effects accounting for 22.47%, 22.52%, and 37.48% of the total effects, respectively, indicating that Qi deficiency constitution, Yang deficiency constitution, and Qi stagnation constitution can not only directly affect CRF in BC patients but also indirectly affect CRF through sleep quality. Therefore, the mediating effect of sleep quality should be fully considered. Nevertheless, given that patients focus on cancer itself and do not actively report their sleep, clinical health care personnel often neglect the assessment of sleep quality in BC patients (41).

In future clinical work, medical personnel should take the initiative to ask BC patients about their sleep status, monitor it, assess sleep function reasonably, identify the causative and related factors of sleep disorders, and provide appropriate guidance and interventions to improve sleep quality. Qi deficiency, Yang deficiency, and Qi stagnation constitutions are categorized as unbalanced constitutions. BC patients predominantly exhibiting unbalanced constitutions demonstrate a greater propensity for disease recurrence and metastasis, poorer prognosis, and elevated levels of CRF (42). Medical personnel should formulate personalized care plans for BC patients, pay attention to the TCM constitution of BC patients, monitor the health status of BC patients with unbalanced constitutions at any time, consider individual differences, and effectively apply “TCM Constitution Identification” to implement health management.

## 5 Limitations

This study has several limitations. First, its cross-sectional design cannot establish causal relationships among TCM constitution, sleep quality, and CRF. Second, the use of convenience sampling to recruit patients from two Grade A tertiary hospitals in the same city may introduce selection bias, limiting the generalizability of the findings to a broader population of breast cancer patients, particularly those in other regions or at medical institutions of different levels of care. Future multicenter studies with larger sample sizes are necessary. Third, data were based on self-reported questionnaires, which are subject to recall and social desirability biases. Finally, although the PSQI records the frequency of sleep medication use, it does not include specific drug names, dosages, or other concomitant medications, which constitutes a potential source of confounding.

## 6 Conclusions

The level of CRF in BC patients is moderate, and TCM constitution and sleep quality are important factors that affect CRF. Therefore, nursing staff can reduce the degree of CRF in BC patients by improving their unbalanced constitutions and sleep quality. However, this study has several limitations. First, only BC patients in tertiary hospitals in Jinan city were selected as survey subjects, which has geographical limitations; the survey scope could be expanded to increase the representativeness of the sample in future studies. Second, this study used the highest TCM constitution transformation score to analyze the results. Nevertheless, many mixed constitutions are encountered in the clinical setting. The effects of mixed constitutions on health have not been explored, so future studies can explore the relationships among mixed constitutions, sleep quality, and CRF to validate the results of the present study.

## Data Availability

The raw data supporting the conclusions of this article will be made available by the authors, without undue reservation.
