# Correction: Correlations among traditional Chinese medicine constitutions, sleep quality, and cancer-related fatigue in patients with breast cancer

**DOI:** 10.3389/fpubh.2026.1944933

**Published:** 2026-08-11

**Authors:** Yi Lu, Yanmin Xue, Xiangmin Xu, Zhongning Zhou, Xiyu Wang, Yingtao Meng, Mingxia Li

**Affiliations:** 1 Nursing School, Shandong University of Traditional Chinese Medicine, Jinan, Shandong, China; 2 Cancer Hospital of Shandong First Medical University Shandong Cancer Institute, Jinan, China

**Keywords:** breast neoplasms, fatigue, mediation analysis, medicine, Chinese traditional, sleep quality

Author Xiangmin Xu was omitted as an author in the published article. The correct author list reads:

Yi Lu, Yanmin Xue, Xiangmin Xu, Zhongning Zhou, Xiyu Wang, Yingtao Meng and Mingxia Li

Xiangmin Xu's affiliation is “Nursing School, Shandong University of Traditional Chinese Medicine, Jinan, Shandong, China”.

The **author contributions** statement was erroneously given as:

YL: Investigation, Writing – original draft, Formal analysis, Software, Project administration, Conceptualization, Data curation, Methodology, Writing – review & editing, Supervision. YX: Methodology, Visualization, Investigation, Data curation, Validation, Writing – review & editing, Formal analysis, Conceptualization. ZZ: Software, Methodology, Writing – review & editing. XW: Supervision, Validation, Software, Writing – review & editing. YM: Writing – review & editing, Funding acquisition. ML: Software, Project administration, Funding acquisition, Writing – review & editing, Validation, Supervision.

The correct **author contributions** statement is:

YL: Investigation, Writing – original draft, Formal analysis, Software, Project administration, Conceptualization, Data curation, Methodology, Writing – review & editing, Supervision. YX: Methodology, Visualization, Data curation, Validation, Writing – review & editing, Formal analysis, Conceptualization. XX: Investigation, Data curation, Writing – original draft. ZZ: Software, Methodology, Writing – review & editing. XW: Supervision, Validation, Software, Writing – review & editing. YM: Writing – review & editing, Funding acquisition. ML: Software, Project administration, Funding acquisition, Writing – review & editing, Validation, Supervision.

In the **Acknowledgements**, the section did not disclose that an earlier version of the research reported in the article had appeared in Xiangmin Xu's master's thesis, and the corresponding thesis reference (43) was omitted. The thesis has now been cited and inserted in the Acknowledgments section.

The corrected **Acknowledgments** section reads:

“We would like to thank all of the patients who participated in the study. We also acknowledge that an earlier version of the research reported in this article appeared in Xiangmin Xu's master's thesis (43).”

**Reference**

43. Xu X. *Study on the correlation between TCM constitution, sleep quality, and cancer-related fatigue in patients with breast cancer* [master's thesis] [in Chinese]. Shandong University of Traditional Chinese Medicine, Jinan (2023). doi: 10.27282/d.cnki.gsdzu.2023.000944

The original version of this article has been updated.

